# Advancing mesenchymal stem cell therapy for kidney diseases in companion animals: from mechanisms to clinical application

**DOI:** 10.3389/fvets.2026.1771337

**Published:** 2026-03-27

**Authors:** Yu Yu, Ying Wang, Yunpeng Mu, Siyu Wang, Xuping Zhang, Yizhe Song

**Affiliations:** 1 College of Life Sciences, Yantai University, Yantai, Shandong, China; 2 Department of Otorhinolaryngology, Yantaishan Hospital, Yantai, Shandong, China

**Keywords:** AKI, CKD, companion animals, delivery, homing, mesenchymal stem cells, preconditioning

## Abstract

Acute and chronic kidney diseases are major clinical challenges in companion animals, yet therapeutic options for reversing established injury remain limited. Mesenchymal stem cells (MSCs) have emerged as a promising therapy due to their immunomodulatory and tissue-repair properties. This review synthesizes current evidence on MSC therapy for kidney disease in cats and dogs, with a focus on mechanisms, preconditioning strategies, delivery routes, and clinical outcomes. Key findings indicate that MSCs exert renoprotective effects primarily through paracrine-mediated immunomodulation rather than direct differentiation. Intravenous administration, while simple, results in >80% pulmonary entrapment and renal homing below 5%; arterial or local injection increases homing to 15–20% but carries procedural risks and lacks standardized dosing. Preconditioning strategies (hypoxia, melatonin, ATRA) enhance MSC survival and homing in rodent models, but feline- and canine-specific validation remains limited. Clinical data from 19 studies in cats/dogs demonstrate that MSC therapy is generally safe, with most adverse events being infusion-related and transient. In feline CKD, consistent trends toward improved glomerular filtration rate and quality of life are reported, although statistical significance is rarely achieved. In canine AKI, MSC therapy improves survival and renal function, but results are heterogeneous and predominantly derived from experimentally induced models. Major evidence gaps include lack of dose standardization, variable MSC characterization, short follow-up durations, and limited long-term safety data. Moving forward, establishing consensus on weight-based dosing, MSC characterization standards, and multicenter randomized controlled trials in naturally occurring disease is essential to translate preclinical promise into clinical practice.

## 1 Introduction

Kidney disease is a major global health burden affecting human populations and ranks among the top 10 causes of mortality worldwide, with its incidence continuously rising. Annually, approximately 13.3 million new cases of acute kidney injury (AKI) occur in humans, contributing to an estimated 1.7 million human deaths (1). According to the Kidney Disease: Improving Global Outcomes consensus, renal pathologies are classified as either acute kidney injury or chronic kidney disease (CKD) based on the severity, reversibility, and temporal onset of functional impairment (2, 3). Spontaneous kidney disease is highly prevalent in companion animals, particularly cats and dogs, and closely mimics the pathological features observed in human kidney disorders (4, 5). In recent years, the global companion animal market continues to expand, driving growing demand for effective treatments for kidney disease. Yet veterinarians still face limited therapeutic options and the challenge of reversing established renal impairment.

Currently, effective disease-modifying therapies for acute kidney injury (AKI) remain lacking. Current standard management adheres to a five key principle framework, encompassing early warning, identification, screening, intervention, and prognostic assessment (6–8). These conventional interventions primarily aim to prevent further renal deterioration and capitalize on the golden window for functional recovery; however, they fail to substantially reverse established renal parenchymal injury or adequately modulate the dysregulated immune responses involved in the pathogenesis of AKI (9, 10). Similarly, the management of chronic kidney disease continues to rely on blood pressure control, glycemic management, statin therapy, and dietary modification (11, 12). Although these strategies mitigate disease progression by reducing metabolic risks, they do not fundamentally repair renal parenchymal damage (3, 13, 14). With the advancement of regenerative medicine, the emergence of cell-based therapies offers new promise for renal repair.

Mesenchymal stem cells, characterized by their self-renewal capacity, multilineage differentiation potential, and potent regenerative and immunomodulatory properties, represent a promising therapeutic approach for kidney diseases (15, 16). Their therapeutic effects are primarily mediated by anti-inflammatory activity, immunoregulation, and tissue regeneration (17–19). MSCs exert anti-inflammatory effects primarily through paracrine signaling, secreting factors such as IL-10 that suppress pro-inflammatory mediators like TNF-α and IL-6 (20–22). Concurrently, they secrete growth factors (e.g., HGF, VEGF-C) and immunomodulatory cytokines (e.g., TGF-β), which mediate immune cell regulation—notably promoting macrophage polarization toward the M2 phenotype (17, 23, 24). While MSCs can engraft into injury sites and differentiate into tissue-specific cells, this direct regenerative mechanism contributes minimally to overall efficacy compared to paracrine actions (25–27). These mechanisms have shown promise in canine and feline kidney disease models, with MSCs demonstrating favorable outcomes across multiple therapeutic programs (28–30).

However, MSC-based therapies have shown inconsistent therapeutic outcomes. To enhance post-infusion efficacy, research has converged on three key strategies: cellular preconditioning, delivery route optimization, and carrier-assisted delivery (31–33). Cellular preconditioning involves modifying MSCs during *in vitro* culture to augment their therapeutic potential. This includes pharmacological preconditioning with bioactive compounds and genetic engineering approaches (e.g., targeted gene knockdown or overexpression) to enhance specific functional properties (34, 35). Delivery route optimization aims to maximize renal homing while minimizing off-target sequestration. Beyond standard peripheral intravenous infusion—which subjects cells to significant pulmonary first-pass effects—investigated approaches include direct renal artery infusion, intraparenchymal renal injection, and subcapsular delivery (33, 36). Carrier-assisted delivery leverages advances in biomaterial science. Engineered carriers (e.g., hydrogels, microspheres) protect MSCs during transit, enhance site-specific retention, and improve homing efficiency without compromising renal safety (31, 32, 37).

**Figure 1:**
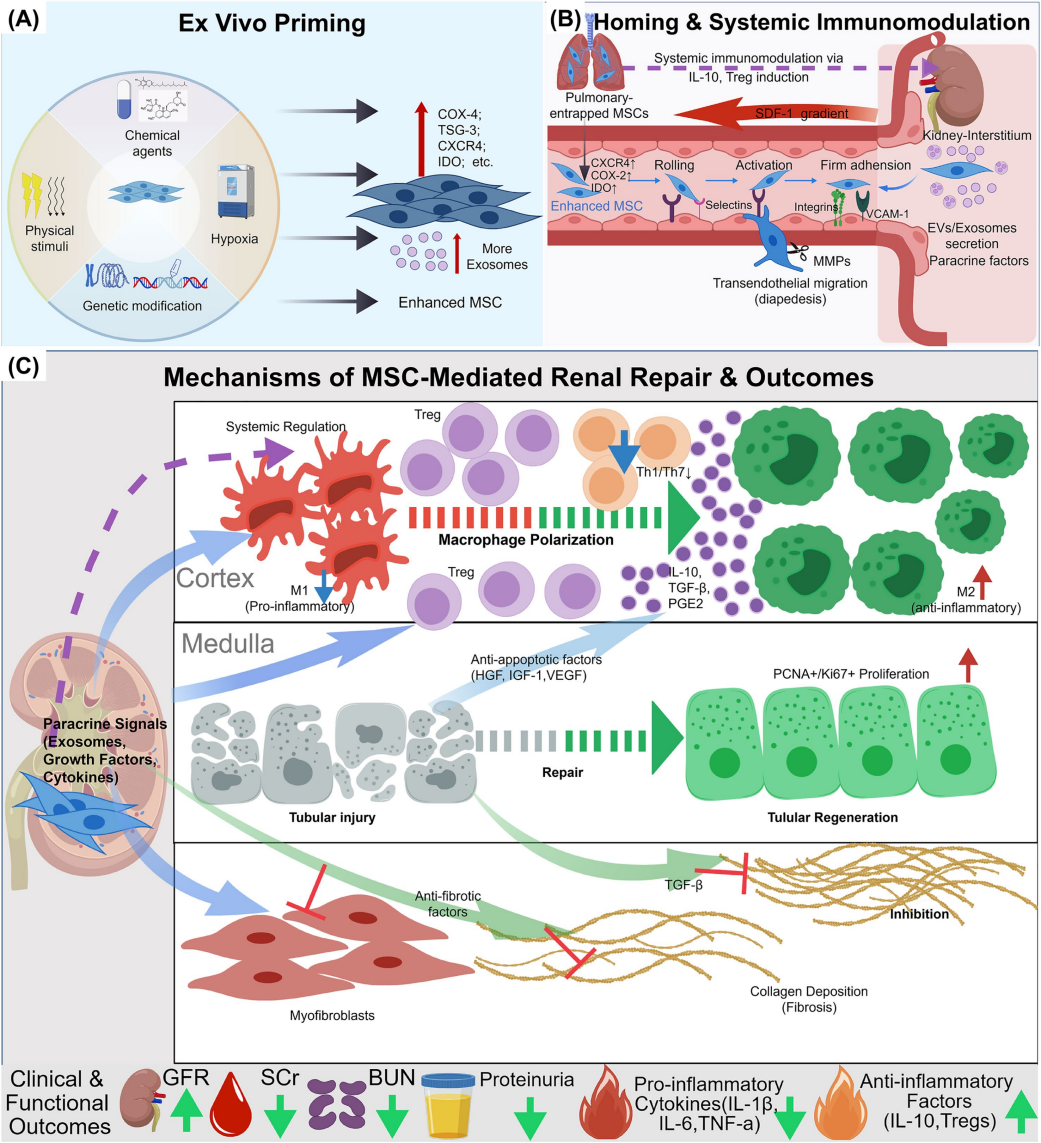
The “Prime–Target–Carrier” framework for MSC therapy in kidney disease. This figure illustrates three core strategies for optimizing MSC-based renal therapy. **(A)** *Ex Vivo* Priming. MSC are enhanced via four priming strategies: (1) chemical agents (ATRA, melatonin, atorvastatin); (2) physical stimuli (hypoxia, laser, electromagnetic fields); (3) genetic modification (Klotho, HGF, ISL1, CXCR4 overexpression); and (4) hypoxic preconditioning (1% O₂). Primed MSCs upregulate functional molecules (COX-4, TSG-3, CXCR4, IDO), enhance paracrine activity, increase EV/exosome production, and become “enhanced MSCs.” **(B)** Homing and Systemic Immunomodulation. Following IV infusion, >80% of MSCs are lung-entrapped, while <5% home to the kidney via SDF-1/CXCL12 gradient. Homing involves: (1) CXCR4/COX-2/IDO-mediated enhancement; (2) selectin-mediated rolling; (3) integrin (α4β1, α5β1)-VCAM-1 adhesion; and (4) MMP-mediated transendothelial migration. Lung-entrapped MSCs exert systemic effects via IL-10 and Treg induction. Renal interstitial MSCs secrete EVs/exosomes and paracrine factors (HGF, VEGF, IL-10, PGE₂) to promote repair. **(C)** Renal Repair and Outcomes. MSCs promote kidney repair via three pathways: (1) Immunomodulation: M1 → M2 macrophage polarization, Treg expansion, Th1/Th17 suppression; (2) Tubular regeneration: PCNA^+^/Ki67^+^ epithelial cell proliferation via paracrine/anti-apoptotic factors (HGF, IGF-1, VEGF); (3) Anti-fibrosis: Inhibition of α-SMA^+^ myofibroblasts and TGF-β-mediated collagen deposition. Outcomes include improved GFR, reduced SCr/BUN/proteinuria, decreased pro-inflammatory cytokines (IL-1β, IL-6, TNF-α), and increased anti-inflammatory mediators (IL-10, Tregs). Created with BioGDP.com.

This review synthesizes research progress on mesenchymal stem cell therapy for kidney diseases, emphasizing how delivery routes and pre-treatment strategies influence cell homing and therapeutic efficacy. It examines existing challenges based on current clinical applications in cats and dogs, and outlines future research directions. By examining the literature through an integrated framework of pre-treatment enhancement, targeted delivery, and vehicle-assisted strategies (Figure 1), we assess the current evidence base. This framework aims to support clinical practice in companion animal nephrology and lays the groundwork for optimizing future veterinary trial designs.

Although this is not a formal systematic review, we conducted a structured literature search for the section on clinical applications in companion animals (Chapter 6) to ensure comprehensive coverage. We searched PubMed, Web of Science, and the Cochrane Library from January 1, 2015, to February 1, 2026, using the following keyword combinations: (“mesenchymal stem cells” OR “MSC”) AND (“acute kidney injury” OR “chronic kidney disease”) AND (“dog” OR “canine” OR “cat” OR “feline” OR “companion animal”). The search included studies examining MSC transplantation from any source in dogs and cats with experimental or naturally occurring AKI or CKD, using intravenous, intra-arterial, or parenchymal administration. Exclusion criteria were narrative reviews, observational studies, editorials, commentaries, and conference abstracts; only English-language articles were included. Retrieved records were compiled in EndNote, and duplicates were removed. Articles were then screened by title, abstract, and keywords; studies not meeting the inclusion criteria were excluded.

## 2 Core mechanisms of mesenchymal stem cell therapy for kidney disease

The pathogenesis and progression of kidney disease involve three interconnected pathological processes: glomerular filtration impairment, tubulointerstitial damage, and progressive renal fibrosis (13, 38–43).

MSCs have emerged as a promising therapeutic strategy for kidney disease, offering distinctive therapeutic advantages (44–46). Their multilineage differentiation potential enables them to differentiate into renal parenchymal cells—including tubular epithelial cells and podocytes—under specific microenvironmental conditions, thereby facilitating the direct structural repair of damaged renal tissues (25–27). Beyond direct tissue repair, MSCs exhibit potent immunoregulatory properties by modulating of the local immune microenvironment. This suppresses excessive immune responses and attenuates renal inflammation (20, 21, 47, 48). Key mechanisms include inhibiting T-lymphocyte proliferation, downregulating pro-inflammatory cytokines (e.g., TNF-α, IL-1β), and upregulating anti-inflammatory cytokines (e.g., IL-10). These immunomodulatory effects have been comprehensively reviewed by Han et al. (49). Furthermore, MSCs induce macrophage polarization toward the M2 phenotype, which is characterized by anti-inflammatory activity and tissue-repair capabilities. As demonstrated by Geng et al., MSC-mediated activation of trophic M2 macrophages promotes functional recovery in rhabdomyolysis-induced AKI by facilitating the transition from tubular injury to regeneration (50).

In a seminal study, Wang et al. demonstrated that MSCs enhance acute kidney injury repair through the dual mechanisms of mitochondrial regulation and ferroptosis suppression (51). Specifically, MSCs upregulated PGC-1α expression in renal tubular epithelial cells, thereby restoring mitochondrial dynamics through balanced modulation of fusion and fission proteins, reducing reactive oxygen species production, and inhibiting NLRP3 inflammasome activation. Concurrently, MSCs administration significantly reduced pyroptosis biomarkers including IL-18 while exerting potent antifibrotic effects. These findings collectively indicate that MSCs not only facilitate acute recovery but also provide long-term protection against fibrotic progression (52).

Although most mechanistic insights are derived from rodent studies, emerging evidence in dogs and cats now supports these pathways. For example, in canine renal ischemia–reperfusion models, ADMSC-derived exosomes have been shown to reduce renal cell apoptosis, mitochondrial damage, and hydrogen peroxide production, thereby attenuating renal injury (46, 53). However, certain mechanisms warrant further investigation specifically in companion animal models.

## 3 Preconditioning strategies and mechanisms of mesenchymal stem cells

To augment the therapeutic efficacy of mesenchymal stem cells, contemporary research employs strategic preconditioning through optimized *in vitro* culture parameters, physical stimulation, genetic engineering, and biochemical modulation via growth factors or pharmaceutical agents (Figure 1).

### 3.1 Physical preconditioning

Optimizing MSC culture conditions has been shown to significantly enhance their proliferation and differentiation capacity. When cultured under hypoxia conditions, MSCs exhibit increased secretion of beneficial factors, attenuated replicative senescence, elevated proliferation rates, enhanced differentiation potential, and greater release of anti-inflammatory cytokine. These changes collectively strengthen their antioxidant and anti-inflammatory capabilities (54–57). Ishiuchi et al. demonstrated that hypoxic preconditioning of MSCs augments their paracrine activity, thereby enhancing renoprotective effects and attenuating renal fibrosis and inflammation in rats with I/R injury (58). Similarly, Zeng et al. reported that hypoxia-treated MSCs mitigate renal tubular cell apoptosis and preserve renal function during rat I/R injury by promoting tissue autophagy. Other physical preconditioning strategies have also been shown to enhance MSCs functionality. Low-intensity laser irradiation elevates proliferative activity (59), while extremely low-frequency pulsed electromagnetic fields accelerate differentiation, stimulate collagen deposition, and amplify anti-inflammatory responses in MSCs (60).

### 3.2 Gene modification of MSCs

Gene modification has emerged as a potent preconditioning strategy to enhance MSC function through the introduction of specific therapeutic genes such as those encoding anti-apoptotic factors or growth factors (61). Zhang et al. transduced mouse bone marrow-derived mesenchymal stem cells (BMSCs) with Klotho-GFP adenovirus and transplanted them into mice with AKI. They demonstrated that Klotho overexpression inhibited the Wnt/β-catenin pathway in renal tubular epithelial cells, significantly enhancing the antifibrotic effects of BMSCs while concurrently improving their proliferative capacity and immunomodulatory function (62). Similarly, Shao et al. reported that hepatocyte growth factor (HGF) modification of dental pulp stem cells markedly attenuated renal fibrosis, potentially via PI3K/AKT/GSK3β pathway-mediated regulation of fibrosis-related genes (63). Wang et al. found that BMSCs overexpressing ISL1 attenuated renal ischemia–reperfusion injury through the paracrine secretion of haptoglobin, thereby exerting anti-apoptotic and antioxidant effects (64). Despite notable progress, the long-term safety and stability of genetically modified MSCs, particularly regarding their potential risks in clinical applications, require further rigorous investigation.

### 3.3 Drug preconditioning

Drug preconditioning has emerged as a promising strategy to enhance MSC therapeutic efficacy. By modulating key cellular pathways, pharmacologically preconditioned MSCs amplify beneficial processes—such as growth factor secretion, anti-inflammatory responses, macrophage polarization, and regulatory T-cell induction, while suppressing detrimental pathways, ultimately improving migration, proliferation, and renoprotection (Table 1) (65–76). Notable examples include the following:

**Table 1:** Effects and principles of pretreating stem cells with drugs.

Name	Principles	Efficacy	References
Iron-quercetin complex	By promoting the expression and secretion of Hepatocyte Growth Factor (HGF), activating the HGF/c-Met signaling pathway, and inhibiting apoptosis of tubular cells	Serum creatinine and blood urea nitrogen levels were significantly reduced, renal tissue structure was improved, and the scores for tubular necrosis and injury were decreased.	(65)
All-trans retinoic acid	Product hyaluronic acid, activate the PI3K/AKT pathway Potentiates the antioxidant effect, enhance its effect on the activation of the Wnt/beta catenin pathway	Enhance the anti- inflammatory, anti- apoptotic, and proliferative repair effects of MSCs	(66, 67)
Valproic acid	Activating the AKT/PI3K and SDF1/CXCR4 pathways	Significantly improved renal function and increased antioxidants	(68)
Interferon gamma	Enhance regulatory T cell induction	Suppresses inflammation, renal tubular interstitial fibrosis, and accumulation of extracellular matrix, and increases VEGF	(69)
Astilbin	Inducing of M2-like phenotype polarization in macrophages through the PTGS2-mediated pathway	Decreased the IL-6 and TNF-α levels and increased the IL-10 levels	(70)
Hyaluronic acid	Inhibit the Wnt/β-catenin signaling pathway	Reducing inflammatory markers, apoptosis markers, and fibrosis markers	(71)
Rapamycin	Pre-activation of autophagy inhibits the mTOR/AKT signaling pathway	Enhance the survival and differentiation rates of transplanted MSCs, and improving renal tissue.	(72)
Erythropoietin	Up-regulated SIRT1 and Bcl-2 expression and down-regulated p53 expression, inhibit apoptosis of MSCs and enhance their homing and anti-inflammatory capabilities.	Lower serum IL-1β and TNF-α levels and higher IL-10 serum level, improved tubular injury significantly	(73)
Melatonin	Reduces the expression of TGF-β1, α-SMA, and TNF-α, increases the expression of E-cadherin. Activate the P-Erk1/2 and P-Akt signaling pathways, and increase the expression of antioxidant enzymes.	Enhance homing ability and survival rate, and improve renal regeneration outcomes	(74, 75)
Atorvastatin	Suppression of TLR4 signaling	Ameliorated oxidative stress, inhibited inflammation response, and increased the viability of implanted MSCs	(76)

Astilbin, a multifunctional flavonoid, protects MSCs from oxidative stress and enhances their antioxidant capacity. In models of AKI-to-CKD transition, astilbin-preconditioned MSCs attenuated AKI/CKD progression through amplified immunomodulation, mediated primarily via PTGS2-dependent macrophage polarization (70).

All-trans retinoic acid (ATRA) boosts MSC efficacy through distinct mechanisms. Zhang et al. reported that ATRA-preconditioned MSCs enhanced hyaluronic acid production and activated renal tubular PI3K/AKT signaling, thereby potentiating AKI recovery (67). Notably, Barakat et al. demonstrated that ATRA-treated Wharton’s jelly MSCs upregulated Wnt/β-catenin signaling while attenuating cell death, inflammation, and oxidative stress (66).

Erythropoietin preconditioning of bone marrow-derived MSCs accelerated AKI repair in rats, significantly reducing serum creatinine, blood urea nitrogen, and histopathological scores compared to untreated MSCs (73). Collectively, these findings confirm drug preconditioning substantially enhances MSC therapeutic outcomes. Future studies should prioritize identifying novel preconditioning agents based on mechanistic synergy with MSC biology and disease pathways.

Regarding pre-treatment-related trade-offs, Boyette et al. reported that hypoxic conditioning of human bone marrow-derived MSCs enhanced their ability to generate highly clonogenic progenitor cells but compromised their differentiation potential (77). In a related review, Hu and Li concluded that pre-treatment often enhances one functional property at the expense of others (78). To date, research on pretreated MSCs has largely focused on *in vitro* phenotypes, secreted factors, and short-term outcomes in animal models. This raises a critical question: Do pre-treatment-modified MSCs differ significantly from unmodified MSCs in terms of long-term efficacy and safety?

In feline and canine MSCs, an *in vitro* study by Park et al. demonstrated that IFN-*γ* pre-treatment of feline adipose-derived MSCs enhances immunomodulatory function through the PGE2 pathway, inducing macrophage polarization and increasing regulatory T cell numbers (79). Separately, Yun, Park, and colleagues used mRNA engineering to overexpress TSG-6 in canine adipose-derived MSCs; co-culture with DH82 macrophages confirmed that TSG-6 overexpression significantly enhanced anti-inflammatory effects (80). These findings align with rodent data; however, *in vivo* studies in cats and dogs with kidney disease remain lacking (Table 2). Future research should prioritize translating pre-treatment strategies validated in rodent models to address this gap.

**Table 2:** Summary of MSC preconditioning strategies tested in renal disease models and their reported effects.

Preconditioning type	Specific method	Animal model	Homing efficiency	Cell survival	Anti-fibrosis	Renal function improvement	References
Physical	Hypoxia (1% O₂)	Rat IRI	↑ (CXCR4)	↑	↑	↑ (SCr/BUN)	(58)
Genetic modification	Klotho overexpression	Mouse AKI	–	↑	↑	↑	(62)
	HGF modification	Mouse fibrosis	–	–	↑	–	(63)
	ISL1 overexpression	Rat IRI	–	↑ anti-apoptosis	–	↑	(64)
	CXCR4 overexpression	Mouse AKI	↑↑	–	–	↑	(94)
Pharmacological	Iron-Quercetin complex	Rat AKI	–	↓ apoptosis	–	↑	(65)
	All-trans retinoic acid	Rat IRI	–	↑	–	↑	(67)
	All-trans retinoic acid	Rat AKI	–	–	–	↑	(66)
	Valproic acid	Rat IRI	↑ (CXCR4)	↑	↓	↑	(68)
	Interferon-γ	Rat IRI	–	–	↑	↑	(69)
	Astilbin	Mouse AKI -CKD	–	–	↑	↑	(70)
	Hyaluronic Acid	Rat fibrosis	–	–	↑	–	(71)
	Rapamycin	Rat AKI	–	↑	–	↑	(72)
	Erythropoietin	Rat AKI	↑ (CXCR4)	↑	–	↑	(73)
	Melatonin	Rat CKD	↑	↑	↑	↑	(74)
	Melatonin	HK-2	–	↑	–	–	(75)
	Atorvastatin	Rat IRI	–	↑	–	↑	(76)
Feline/Canine specific	IFN-γ preconditioning (feline)	*In vitro* (macrophage co-culture)	–	–	–	–	(79)
	TSG-6 overexpression (canine)	*In vitro* (macrophage co-culture)	–	–	–	–	(80)

↑, Improvement or enhancement; ↑↑, Significant enhancement; ↓, Reduction or inhibition; –, Not assessed or not reported; AKI, Acute kidney injury; CKD, Chronic kidney disease; IRI, Ischemia–reperfusion injury; CXCR4, C-X-C chemokine receptor type 4; HGF, Hepatocyte growth factor; IFN-γ, Interferon-γ; TSG-6, Tumor necrosis factor-inducible gene 6 protein.

## 4 Targeting the kidney: from homing mechanisms to delivery outcomes

Cell homing refers to the process by which MSCs migrate from the site of administration to damaged tissues and subsequently reside within them, representing a critical determinant of therapeutic success (81). This process involves not only interactions between cell surface markers and target the tissue, but also regulation by various physicochemical factors (82).

### 4.1 Molecular basis of renal homing: chemokine-mediated cell migration

Cell homing involves multiple molecular mechanisms, particularly chemokine signaling and receptor interactions. The SDF-1/CXCR4 axis is central to this process, directing CXCR4-positive MSCs toward damaged renal tissue (83–85). Integrin family members are also critical for MSCs homing. By recognizing specific extracellular matrix sequences such as the RGD motif, integrins mediate both the physical coupling of MSCs to their microenvironment and the transduction of biochemical signals. Through α4β1 and α5β1 integrins, MSCs form cross-receptor complexes that activate the FAK–PI3K/Akt pathway and induce PDGFR-β phosphorylation, thereby enhancing their migratory capacity. Concurrently, CXCL12/CXCR4 signaling upregulates integrin α5β3 and connexin expression, driving transendothelial migration and tissue colonization to complete therapeutic homing (86–89).

### 4.2 Regulation of homing efficiency by preconditioning

Pre-treatment strategies also play a key role in modulating homing capacity. Hypoxic preconditioning enhances MSC migratory ability by promoting chemokine receptor expression on the cell surface (90, 91). In addition to hypoxic culture, genetic modification and drug pre-treatment further enhance homing by altering the biological behavior of MSCs. For example, MSCs overexpressing CXCR4 exhibit stronger chemotaxis and migrate more effectively to damaged tissue *in vivo* (92, 93). Liu et al. demonstrated that CXCR4-overexpressing BMSCs exhibited markedly increased renal accumulation (94). In a study by Zhou et al., erythropoietin pre-treatment significantly upregulated CXCR4 expression, altered surface molecule profiles, reduced adhesion to pulmonary capillaries, and decreased pulmonary retention. As a result, renal homing of BMSCs was markedly enhanced (73).

### 4.3 Effect of administration route on renal homing efficiency

**Figure 2:**
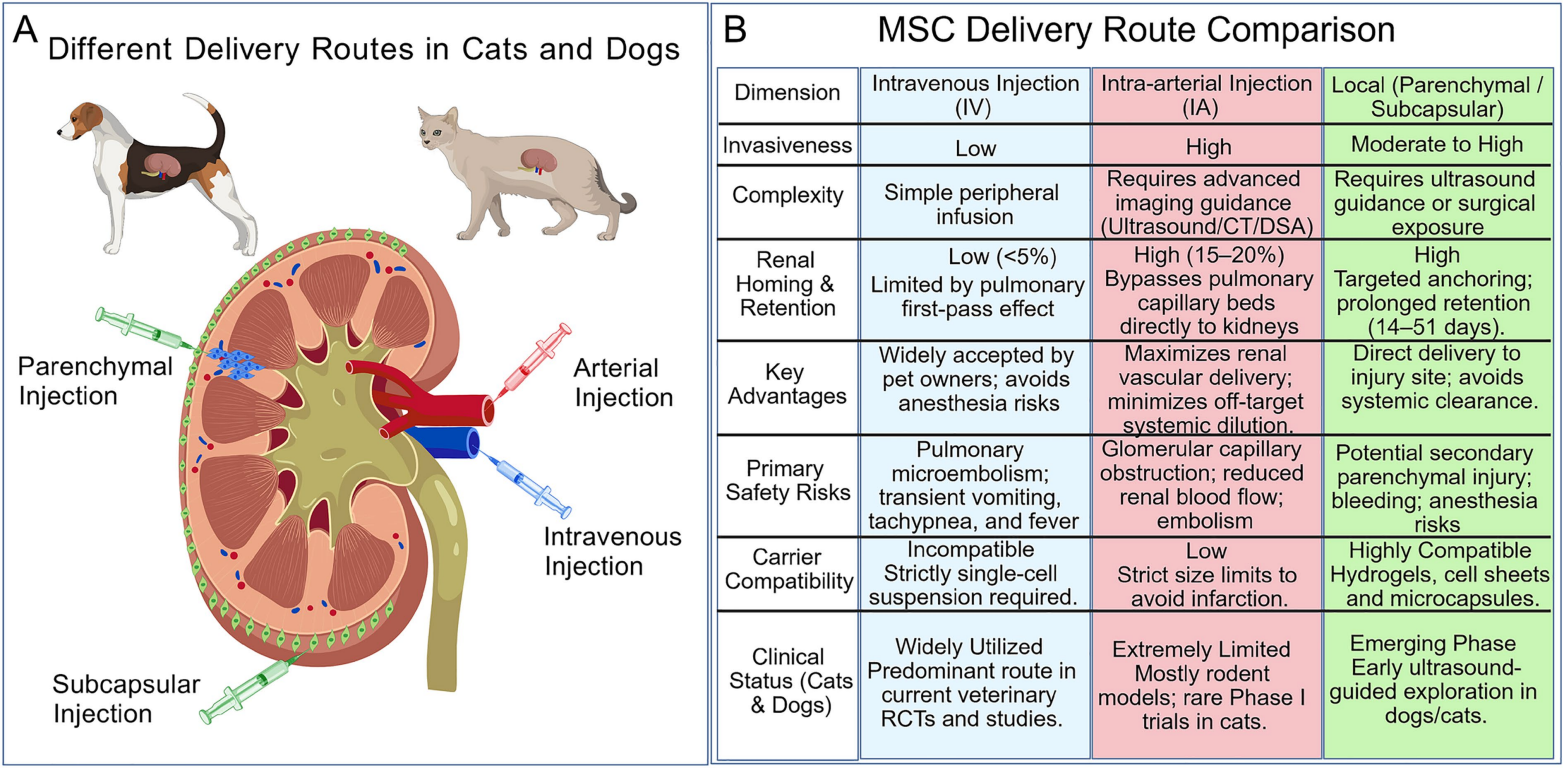
Comparison of MSC delivery routes for kidney disease in companion animals. **(A)** Three principal administration routes for MSC therapy in cats and dogs: intravenous injection, renal artery injection, and renal parenchymal/subcapsular injection. **(B)** Comparison of intravenous injection, intra-arterial injection, and local injection regarding: Invasiveness, Complexity, Renal Homing & Retention, Key Advantages, Primary Safety Risks, Carrier Compatibility, and Clinical Status (Cats and Dogs). Created with BioGDP.com.

The route of administration plays a critical role in determining MSC homing efficiency and therapeutic outcomes (Figure 2). Common administration routes for the treatment of nephropathy include intravenous, intra-arterial, and local injection, each offering distinct characteristics and clinical applications (95, 96). Perirenal approaches, including renal artery, intrarenal, and subcapsular delivery, may offer advantages over peripheral venous infusion as techniques continue to advance (97).

Intravenous administration leads to a significant pulmonary first-pass effect, with the majority of MSCs becoming entrapped in the lungs shortly after injection. While a small fraction distributes to the liver and spleen, only a limited number reach the kidneys or other target organs (36). This pulmonary retention is primarily attributed to several factors: the relatively large size of MSCs compared to the pulmonary capillary diameter, the extensive capillary network in the lungs, and the strong adhesive properties of culture-expanded MSCs (98, 99). In comparative kidney disease studies, untreated MSCs showed renal homing rates below 5% at 24 h post-injection, with 60–80% retained in the lungs and renal persistence lasting approximately 3–5 days. In contrast, pretreated or genetically modified MSCs achieved homing rates of 15–20% (66, 100, 101). Nevertheless, most studies using intravenous administration report that even unmodified MSCs confer measurable renal improvement, with higher homing rates correlating with better outcomes. Functional parameters including serum creatinine and blood urea nitrogen levels, decrease following treatment, and histological examination confirms renal recovery (66, 67, 95). Cell dose also influences pulmonary retention and systemic distribution: high-dose intravenous administration (>1 × 10^6^ cells) has been associated with superior efficacy compared to lower doses (102).

Renal artery injection represents a more direct approach that offers high targeting specificity. By bypassing pulmonary capture, it delivers MSCs directly into the renal vasculature, theoretically enhancing homing efficiency and therapeutic outcomes. However, technical complexity and the risk of embolism limit its widespread application (33, 103–107). Yamada et al. demonstrated that administration of 5 × 10^5^ MSCs via renal artery injection led to stable colonization of the kidneys within 1–3 days, with significantly fewer cells retained in the lungs. In a mouse model of ischemia–reperfusion injury (IRI), tracked MSCs remained detectable in the kidneys up to 42 days post-injection; in contrast, even five times the dose delivered intravenously was undetectable in the kidneys by day 7 (103). In a rat model, Cai et al. showed that renal artery injection of a low dose (1 × 10^5^ cells) yielded superior efficacy compared to tail vein or carotid artery administration of 1 × 10^6^ cells. The same study also revealed that high-dose arterial injection may cause glomerular capillary loop obstruction and reduce renal blood flow (105). Although renal artery injection consistently achieves high homing efficiency and shows therapeutic promise in preclinical models, concerns regarding embolization, procedural complexity, and potential secondary injury during kidney exposure have confined most studies to rodents, with limited evaluation in large animal or clinical settings. Given that the associated risks currently outweigh the potential benefits in veterinary practice, refinement of surgical techniques remains the central challenge for advancing this approach.

Local injection, primarily via the renal capsule or renal parenchyma, enables direct MSC delivery into the kidney. This approach markedly increases MSC retention within renal tissue, thereby improves therapeutic efficacy (108–111). Huang et al. compared these two methods by transplanting umbilical cord-derived MSCs encapsulated in a collagen matrix into AKI-CKD mice via subcapsular and parenchymal routes. Both local MSC therapies reduced tubular injury, promoted tubular epithelial cell proliferation by day 3, and alleviated renal fibrosis by days 14 and 28. Using live imaging to track labeled cells, strong signals were detected in both groups on day 1, increased by day 3, and gradually diminished thereafter; however, signals remained detectable at 14 days post-transplantation (108). In a separate study, Lee et al. used ultrasound-guided renal parenchymal injection of MSCs to treat canine AKI, administering cells on days 6 and 23 after onset. Green fluorescent protein-labeled MSCs remained detectable in renal sections at day 51 (28). Xiao et al. demonstrated that minimally invasive transplantation of MSCs under the renal capsule using ultrasound guidance was safe and effective in miniature pigs; moreover, multiple transplants promoted renal functional recovery more effectively than a single transplant (111).

### 4.4 Correlation between homing efficiency and treatment outcomes

Current evidence indicates a clear correlation between homing efficiency and therapeutic outcomes: higher renal homing rates are associated with more pronounced reductions in biochemical markers (e.g., serum creatinine, blood urea nitrogen) and greater mitigation of renal tissue damage with accelerated repair. However, therapeutic efficacy is not solely contingent on MSC homing. In studies by Barakat et al. and Wang et al., MSCs improved renal function and histology despite limited homing to the kidney (66, 100). This suggests that MSC effects arise from combined local and systemic immunomodulation rather than homing-dependent mechanisms alone. Supporting this, Li et al. recently demonstrated that allogeneic MHC-expressing MSCs enhance immunomodulatory and anti-inflammatory effects by amplifying Treg responses and promoting IL-10 production, thereby conferring renal protection (112). The growing body of work on MSC-derived extracellular vesicles further underscores this point: although efficacy varies, acellular approaches yield therapeutic outcomes comparable to direct MSC treatment, indicating that homing represents only one component of the broader mechanism (113–121).

Several key questions remain to be addressed: (I) Is there a threshold effect-how many cells must persist within the kidney and continuously secrete paracrine factors to trigger effective treatment? (II) What is the relationship between local retention duration and therapeutic efficacy? (III) For animals with CKD, local injection may represent an optimal strategy for maximizing treatment outcomes. However, these patients frequently present with comorbidities that may elevate the risks associated with anesthesia and invasive procedures (122). Striking a balance between maximizing targeted delivery and minimizing procedural risk thus remains a critical translational challenge in veterinary medicine.

In summary, homing efficiency substantially influences therapeutic efficacy. Nevertheless, clinical treatment plans should be individualized based on disease stage, comorbidities, and procedural risk.

### 4.5 Veterinary clinical perspective: bridging the gap between targeted delivery and clinical application in dogs and cats

Studies in rodent and large animal models have demonstrated that renal artery and local injection can increase MSC homing rates below 5% with IV administration to 15–20% or higher. However, clinical research in companion animals has yet to translate these theoretical advantages into practice. Analysis of existing feline and canine clinical studies reveals that IV injection remains overwhelmingly dominant—whether in early exploratory studies or recent randomized controlled trials, the vast majority have employed the IV route (28–30, 53, 123–131). Only a handful of studies have attempted intrarenal injection (132, 133), while IA and subcapsular routes remain unreported in cats and dogs to date.

This disconnect between theory and practice stems from several implementation barriers: (I) Technical barriers: IA and subcapsular injections require imaging guidance (ultrasound, CT, DSA) that remains limited in veterinary clinical settings. (II) Anesthesia risks: Cats and dogs with CKD often present with comorbidities such as cardiac or endocrine disease, yet insufficient data exist to support risk assessment for interventional procedures under general anesthesia. (III) Owner acceptance: Compared to simple IV infusion, invasive procedures carry higher costs and perceived risks, which may influence treatment decisions. (IV) Dosage experience gap: Rodent studies indicate an optimal dosage range for IA administration—too low is ineffective, too high carries embolism risk. However, optimal IA doses for cats and dogs remain unknown, and determining them would require extensive, resource-intensive experimentation.

Nevertheless, rodent data offer important insights for designing feline and canine trials. If a threshold effect exists, that is, a minimum number of homing MSCs required to initiate effective repair, then exploring IA or subcapsular routes provides a strong rationale for treating refractory CKD patients with suboptimal responses to IV therapy. Future research should prioritize establishing safe dosage ranges and standardized protocols for IA and subcapsular administration in large animal models before moving to clinical trials in cats and dogs with naturally occurring disease.

## 5 Carrier-assisted strategy

Even when MSCs reach the kidneys via optimized administration routes, their short survival and limited retention within the injured microenvironment continue to constrain therapeutic efficacy. Carrier-assisted strategies seek to overcome this final barrier by prolonging MSC retention and sustaining functional activity through physical encapsulation or local anchoring within the kidney.

Hydrogels are three-dimensional networks formed from natural or synthetic hydrophilic polymers, offering a range of physicochemical properties (134). As common carriers for MSC delivery, hydrogels fall into two main categories: natural (collagen, gelatin, alginate, hyaluronic acid) and synthetic (PEG, GelMA) (135, 136). Natural hydrogels provide excellent biocompatibility, degradability, and extracellular matrix mimicry, whereas synthetic variants offer tunable mechanical properties, extended retention, and controlled degradation. Functionally, the three-dimensional scaffold protects encapsulated cells, enables sustained release to prolong the therapeutic window, and supports minimally invasive precision delivery via injection (137, 138). In renal applications, hydrogels can fill the subcapsular space to maximize contact area. Local enrichment is achieved through subcapsular or intrarenal injection, bypassing off-target retention in the lungs, liver, or spleen and thereby enhancing renal retention and therapeutic efficacy (139, 140). Najafi et al. used hydrogels to deliver WJ-MSCs and included control groups receiving either no carrier or nitric oxide donors combined with WJ-MSCs. The triple combination of hydrogel, nitric oxide donors, and WJ-MSCs yielded the most favorable biochemical and histological outcomes, while each monotherapy, though effective, was inferior to the combined approach (141).

Cell microcapsules offer a widely used platform for delivering MSCs. Alginate (ionically crosslinked) and chitosan (thermosensitive phase-change) are commonly selected for their biocompatibility, injectability, and controlled degradation—safety advantages over PLGA, whose acidic degradation products raise concerns. Chitosan can also be optimized by combining it with natural polysaccharides (142, 143). Functionally, microcapsules can be engineered to avoid embolization or traverse filtration barriers through size tuning. Combined with surface modifications (e.g., RGD for adhesion, PEG for immune evasion), core-shell structures, or imaging tracers, they enable single-cell encapsulation, controlled factor release, cell protection, and real-time tracking (144–147). In renal therapy, microencapsulation is frequently paired with local injection. Huang et al. delivered collagen matrix-encapsulated MSCs via local injection in an IRI model and observed tubular repair, with MSCs still detectable in renal tissue at 28 days (108). However, the study lacked an unencapsulated MSCs control group, making it difficult to determine whether therapeutic effects derived from the injection method itself or encapsulation. Future studies should include appropriate controls to clarify this.

MSCs cell sheet technology is a scaffold-free tissue engineering approach that uses thermosensitive polymers such as poly (N-isopropylacrylamide). Compared to conventional methods, this technique better preserves cell surface proteins and maintains functional potency (148–150). MSCs from various sources can be used; however, they exhibit source-dependent differences in adhesion, proliferation, and cytokine secretion. Nakao et al. reported that AD-MSCs secreted higher levels of HGF, whereas BM-MSCs and UC-MSCs showed greater PGE_2_ and IL-6 expression (151). Functionally, the intact ECM network within cell sheets enables seamless integration with host tissue. In an IRI rat model, Ju et al. demonstrated that MSC sheet transplantation significantly reduced BUN and serum creatinine levels, improved tubular injury scores, and inhibited fibrosis progression. *In vivo* imaging confirmed that, compared to single-cell suspension injection, MSC sheets exhibited markedly prolonged renal retention (>14 days) while avoiding systemic complications such as pulmonary embolism (152).

Despite the potential of carrier techniques to improve renal retention in experimental models, their application in clinical practice for companion animals encounters numerous practical challenges. First, cost and complexity: the preparation of synthetic hydrogels and associated surgical procedures raise treatment costs, potentially exceeding what some owners can afford. Second, regulatory pathways remain unclear, with no established approval standards for cell–biomaterial combination products in veterinary medicine. Third, owner acceptance remains a critical variable—for pets with advanced CKD, owners may prefer simpler, low-risk intravenous infusions over invasive procedures requiring anesthesia. Future research should therefore focus on developing injectable, minimally invasive delivery systems, while incorporating owner preference analysis and cost–benefit assessments into clinical trial study designs.

## 6 Clinical applications in companion animals

The MSC treatment strategy described earlier has been shown to enhance homing and amplify therapeutic effects in animal models. However, its clinical evidence in naturally occurring companion animals has yet to be systematically reviewed. Following the literature search and organization described in the introduction, this chapter presents a comprehensive review of existing clinical studies on MSC therapy for kidney diseases in cats and dogs, thereby establishing an evidence base for subsequent translational research (Table 3). A total of 208 articles were initially identified. After removing duplicates and studies that did not meet the inclusion criteria, 19 articles were retained: 11 involving cats and 8 involving dogs.

**Table 3:** Summary of feline and canine clinical studies on MSC therapy for kidney disease.

Species	Indication	Cell source	Dose (cells)	Route	Design	Follow-up	Main outcomes	Adverse events	References
Cat	AKI model	ADMSC/Allo, BMSC/Allo	4 × 10^6^	IV	Controlled study	6 days	Noimprovement in renal function or histopathology	Transient -fever (multiple)	(130)
Cat	AKI model	ADMSC-EVs/Allo	Equivalent to 10^7^ cells/24 h secretion	IV	Case series	3 days	Significantly accelerated creatinine and BUN recovery; identified 6 novel biomarkers	None	(125)
Cat	CKD	BMSC/Auto	1 × 10^5^ cells/kidney (3 sites)	Intrarenal	Case series	30 days	Modest GFR and creatinine improvement in 2/4 early CKD cats (not statistically significant)	Transient microscopic hematuria (in healthy cat)	(132)
Cat	CKD	ADMSC/Allo	2 × 10^6^ (cryopreserved)	IV	Sequential study (1)	90 days	Statistically significant but clinically modest creatinine decrease	None	(128)
			4 × 10^6^ (cryopreserved)		Sequential study (2)		No significant improvement in renal function	2/5 vomiting, 4/5 tachypnea, 1/5 severe dyspnea	
			4 × 10^6^ (culture-expanded)		Sequential study (3)		No significant improvement in renal function	None	
Cat	CKD	ADMSC/Allo	2 × 10^6^/kg	IV	Randomized controlled trial	60 days	No significant creatinine decrease; Quality of life trend improvement	Transient vomiting	(129)
Cat	CKD	Amniotic MSC/Allo	2 × 10^6^	IV	Non-randomized controlled	60 days	Significant creatinine decrease (p = 0.028); UPC decrease (NS)	1/9 vomiting during injection	(30)
Cat	CKD	Uterine MSC/Allo model	3 × 10^7^	IV	Controlled study	182 days	Significant GFR increase (*p* < 0.05 at multiple time points); increased food/water intake; met primary endpoint	1 propofol overdose (unrelated)	(131)
Cat	CKD	BMSC/Auto	1 × 10^6^	Renal artery	Phase I trial	6 months	Safe and feasible; no significant GFR improvement	No serious AEs	(153)
Cat	CKD	ADMSC/Allo	2 × 10^6^/kg × 3	IV	Case report	180 days	IRIS stage IV → II; improved body condition and clinical parameters	None	(29)
Dog	AKI model	cUCB-MSC/Allo	1 × 10^6^/kidney × 2	Renal parenchymal	Controlled study	51 days	100% survival (vs 12.5% control); significant BUN/SCr decrease; histological improvement	NR	(28)
Dog	AKI model	BMSC/Auto	1 × 10^6^/kg	IV	Controlled study	4 days	Worsened renal function (higher BUN/SCr); but reduced histological inflammation/fibrosis	NR	(154)
Dog	AKI model	BMSC/Auto vs. ADMSC/Auto	5 × 10^6^, 10 × 10^6^, 15 × 10^6^	Renal cortical	Dose-escalation	3 months	ADSC superior to BMSC; optimal dose 10 × 10^6^; faster anti-inflammatory effect with ADSC	NR	(127)
Dog	CKD	ADMSC/Allo	1 × 10^6^/kg × 3	IV	Case series	~153 days	Safe; 1/5 dog creatinine decreased (3.5 → 2.4 mg/dL); weight stable/increased	None	(126)
Dog	CKD model	Camel WJ-MSC/Xeno	5 × 10^6^ × 2	Intrarenal	Controlled study	12 weeks	Significant BUN/SCr decrease; IRIS stage III → II; tubular regeneration on histology	NR	(133)

ADMSC, Adipose-derived mesenchymal stem cell; AKI, Acute kidney injury; Allo, Allogeneic; Auto, Autologous; BMSC, Bone marrow-derived mesenchymal stem cell; BUN, Blood urea nitrogen; CKD, Chronic kidney disease; cUCB-MSC, Canine umbilical cord blood-derived mesenchymal stem cell; EV, Extracellular vesicle; GFR, Glomerular filtration rate; IRIS, International Renal Interest Society; IV, Intravenous; MSC, Mesenchymal stem cell; NR, Not reported; NS, Not significant; eRCT, Randomized controlled trial; SCr, Serum creatinine; UPC, Urine protein-to-creatinine ratio; WJ-MSC, Wharton’s jelly-derived mesenchymal stem cell; Xeno, Xenogeneic; Study design notes, Induced models (surgical/pharmacological) are specified in the “Indication” column (AKI model, CKD model). Naturally occurring cases are indicated by CKD/AKI without “model” specification.

### 6.1 Clinical research Progress on feline MSC therapy for kidney disease

In feline kidney disease research, clinical studies have predominantly focused on chronic kidney disease (CKD), whereas investigations into acute kidney injury (AKI) remain relatively limited. In a study by Rosselli et al., 15 cats were divided into three groups (*n* = 5 per group) and received intravenous injections of 4 × 10^6^ ADMSCs, BMSCs, or fibroblasts 1 h after renal reperfusion. Analysis 6 days post-injection revealed that none of the three treatment improved renal function parameters or histopathological outcomes in cats with acute ischemic kidney injury (130). In a study by Li et al., three cats with post-renal acute kidney injury (PR-AKI) were treated with extracellular vesicles derived from feline adipose-derived mesenchymal stem cells (ADMSC-EVs) at a dose equivalent to the amount secreted by 10 million feline ADMSCs over 24 h. After 3 days of treatment, ADMSC-EVs were found to promote rapid recovery in cats with PR-AKI, significantly accelerating the restoration of creatinine and blood urea nitrogen levels. In addition, the study identified six novel PR-AKI biomarkers (carnitine, maltose, D-glucosamine, cytidine, dihydro-lactobionic acid, and stachyose), which could serve as auxiliary indicators for assessing disease progression and therapeutic response to ADMSC-EVs (125).

A total of nine studies have investigated MSC therapy for CKD in cats (29, 30, 104, 123, 128, 129, 131, 132, 153). In an early study, Quimby et al. performed ultrasound-guided unilateral renal injections of MSCs in six cats. Ultrasound-guided intra-renal injection of autologous adipose-derived MSCs was found to be safe and feasible in cats with CKD. Two cats with early-to-mid-stage CKD exhibited moderate improvements in glomerular filtration rate and serum creatinine levels, although these changes were not statistically significant (132). In three consecutive pilot studies, the same group administered intravenous allogeneic adipose-derived MSCs to 21 cats with naturally occurring CKD. Low-dose cryopreserved ADMSCs (2 × 10^6^) demonstrated a favorable safety profile. In contrast, high-dose cryopreserved ADMSCs (4 × 10^6^) were associated with a high incidence of adverse reactions: vomiting occurred in 2 of 5 cats, increased respiratory rate in 4 cats, and severe respiratory distress requiring emergency intervention in 1 cat. Cultured and expanded ADMSCs (4 × 10^6^) also showed good safety. However, none of the treatment outcomes reached statistical significance (128). A randomized placebo-controlled trial involving intravenous infusion of allogeneic adipose-derived MSCs reported no significant improvement in serum creatinine levels, although an upward trend in quality-of-life indicators was observed (129). In a case report, intravenous administration of allogeneic adipose-derived MSCs for CKD led to notable improvements in the affected cat’s body condition score and physiological parameters, with the cat’s IRIS stage reduced from stage IV to stage II (29).

Recently, research has expanded to include other cell sources. One study employed amniotic-derived pluripotent cells for intravenous therapy in cats with chronic kidney disease (CKD), resulting in a significant decrease in serum creatinine (*p* = 0.028), a reduction in the urine protein-to-creatinine ratio (*p* = 0.131), and clinically relevant improvement in urine specific gravity, although the latter did not reach statistical significance (30). Another study used intravenous infusion of uterine-derived allogeneic MSCs to treat cats with nephrectomy-induced CKD, demonstrating that the rate of renal function decline was slower in the treatment group compared to controls (131).

### 6.2 Clinical research progress on canine MSC therapy for kidney disease

In canine acute kidney injury (AKI) research, Lim et al. administered cisplatin to induce AKI in six Beagle dogs. Three dogs received intravenous autologous BMSCs (1 × 10^6^ cells/kg), while the control group received saline injection. After 4 days, biochemical analysis revealed that the BMSC-treated group showed no improvement in renal function and, in fact, exhibited more severe renal injury markers (IRIS stage: control group stage II vs. BMSC group stage III). Histopathological analysis demonstrated a significant reduction in mononuclear inflammatory cell infiltration and fibrosis (*p* = 0.016 and *p* = 0.029, respectively), although no significant differences were observed in other parameters (154). Lee et al. established a gentamicin- and cisplatin-induced kidney injury model in 11 dogs. Three dogs received bilateral intraparenchymal injections of 1 × 10^6^ cells per kidney per dose on days 6 and 23, whereas eight dogs received PBS injections. By day 51, only one dog (12.5%) in the control group survived, compared to all dogs in the treatment group. Biochemical analysis showed significant improvements in blood urea nitrogen (BUN) and serum creatinine (SCr) levels in the treatment group relative to controls (*p* = 0.0485 and *p* = 0.0121). Histopathological evaluation further confirmed more pronounced tissue-level improvements in the treatment group (28). In a more comprehensive study by Osman et al., 60 dogs were allocated into groups and treated with BMSCs or ADMSCs via renal cortical injection at low, medium, and high doses (5 × 10^6^, 10 × 10^6^, and 15 × 10^6^ cells), with treatment durations of 2 weeks, 2 months, and 3 months. ADMSCs demonstrated superior overall efficacy compared to BMSCs. In terms of long-term renal function recovery, BMSCs achieved 8–28% recovery, whereas ADMSCs reached 20–40%. Regarding anti-inflammatory effects, ADMSCs acted more rapidly (1 month vs. 3 months). Histologically, ADMSCs initiated regenerative processes and reduced tissue damage at early time points. The optimal injection dose was identified as 10 × 10^6^ cells (127).

Research on MSC therapy for canine chronic kidney disease (CKD) remains relatively limited but has shown promising therapeutic potential. A case series reported the treatment of five dogs with CKD via intravenous infusion of allogeneic ADMSCs, which was found to be safe and associated with improvements in selected renal function parameters (126). Another innovative study employed allogeneic MSCs derived from camel Wharton’s jelly, administered via intrarenal injection in canine CKD models. Compared to the control group, significant improvements were observed in BUN and SCr levels (*p* < 0.05), along with an increased proportion of regenerated tubules and maintained normal glomerular morphology (133).

### 6.3 Summary of efficacy and safety

Based on the clinical studies in cats and dogs reviewed above, the efficacy and safety of MSC therapy for kidney disease can be summarized as follows:

Efficacy. Although most studies on feline chronic kidney disease (CKD) did not achieve statistical significance, they consistently demonstrated positive trends—including moderate improvements in glomerular filtration rate and serum creatinine levels, upward trends in quality-of-life indicators, and reductions in IRIS stage (29, 129, 132). Studies using alternative cell sources, such as amniotic membrane- or uterus-derived MSCs, have also reported significant decreases in serum creatinine or a slowed decline in renal function (30, 131). In canine acute kidney injury (AKI) studies, the majority of findings indicate that MSC therapy improves survival rates, reduces blood urea nitrogen and serum creatinine levels, and attenuates renal tissue damage (28, 127). However, conflicting results have also been reported (154). Notably, adipose-derived MSCs may offer superior efficacy compared to bone marrow-derived MSCs, with an optimal dose of approximately 10 × 10^6^ cells identified in one study (127). Although research on MSC therapy for canine CKD remains limited, preliminary findings suggest potential benefits in improving renal function (126, 133).

Safety. Most studies indicate that MSC therapy is generally well tolerated. The primary adverse reactions observed are infusion related events, including vomiting, tachypnea, and fever (128, 130). High-dose cryopreserved MSCs (4 × 10^6^ cells) may be associated with an increased risk of such reactions (128). While serious adverse events are rare, the potential risk of pulmonary microembolism warrants continued vigilance. Importantly, long-term safety data beyond 1 year post-treatment remain entirely lacking. In addition, many studies failed to report or control for concomitant therapies (e.g., ACE inhibitors, phosphate binders, dietary management), which may confound the assessment of MSC efficacy.

In summary, existing evidence supports the short-term safety of MSC therapy in feline and canine kidney disease, with encouraging signals of efficacy that warrant further validation through larger, more rigorously designed clinical trials. Studies on feline CKD have primarily relied on naturally occurring cases, providing relatively abundant but small-sample evidence. In contrast, research on canine AKI has largely employed experimentally induced models, which offer high internal validity but require caution when extrapolating findings to clinically ill dogs.

## 7 Challenges and outlook

Despite the demonstrated anti-inflammatory, anti-apoptotic, and pro-reparative effects of mesenchymal stem cells in both AKI and CKD models, their clinical translation remains constrained by several key factors. To address these challenges systematically, future research should adopt a multi-layered strategy structured along temporal dimensions and problem domains, ultimately forming a closed-loop “problem–solution” framework.

### 7.1 Short-term bottlenecks: standardization development and data improvement

#### 7.1.1 Lack of dose and cell standardization

Current MSC research in cats and dogs employs a wide range of cell doses (from 1 × 10^6^ to 3 × 10^7^ cells), with most studies using fixed cell numbers rather than weight-adjusted doses. This inconsistency precludes direct comparison of results. Furthermore, studies exhibit considerable heterogeneity in MSC identification criteria—including viability, surface marker expression, and sterility—which severely undermines research reproducibility. Consequently, establishing consensus on weight-based dosage ranges and defining minimal characterization standards for MSC products is essential. These standards should include viability >90%, CD90/CD105 positivity >90%, CD34/CD45 negativity <2%, absence of bacterial, mycoplasma, or endotoxin contamination, and confirmed tri-lineage differentiation capacity. In addition, reporting cell passage number, culture conditions (including fetal bovine serum status), and cryopreservation and thawing protocols is critical to improving data comparability.

#### 7.1.2 Optimizing clinical trial design

Most studies are limited by small sample sizes (<50 cases), short follow-up periods (<1 year), and a lack of standardized endpoints, making cross-study comparisons difficult (155). Therefore, clinicians should consider conducting multicenter randomized controlled trials (RCTs) that stratify enrollment by IRIS stage and compare the efficacy of MSC transplantation—administered via different infusion routes—against placebo in client-owned cats and dogs with naturally occurring nephropathy. The primary endpoint should extend beyond changes in serum creatinine to include more clinically meaningful outcomes, such as survival time and quality of life. This approach would generate high-quality evidence on therapeutic efficacy to inform veterinary practice and establish a foundation for subsequent dose optimization studies.

### 7.2 Mid-term challenges: mechanism deepening and technical optimization

#### 7.2.1 In-depth research on delivery inefficiencies and homing effects

The renal homing rate of MSCs after intravenous injection is typically low, yet improvements in renal function are still observed (33, 35). This phenomenon suggests that the therapeutic effects of MSCs may be mediated by both local actions and systemic immune regulation, although the relative contribution of each remains unclear. This ambiguity hinders the rational design of administration regimens. Quantitative correlation analysis in large animal models is required to concurrently track cellular distribution and systemic immune markers (e.g., regulatory T cells, IL-10) to clarify the mechanisms underlying homing efficiency. If systemic immune modulation proves predominant, simplified and safer intravenous administration may represent a more practical approach.

#### 7.2.2 Cellular heterogeneity and efficacy decline

Cellular heterogeneity represents another critical bottleneck. Single-cell sequencing confirms significant differences in secretion profiles, homing capacity, and differentiation propensity between different tissue sources (UC-MSC vs. BM-MSC) and subpopulations (e.g., pro-inflammatory vs. immunoregulatory) (156, 157). Furthermore, replicative senescence induced by *in vitro* expansion diminishes MSC stemness, particularly in autologous MSCs from elderly donors, which exhibit reduced proliferative potential and accumulated DNA damage (158). Therefore, single-cell transcriptomics and functional potency assays (e.g., T-cell proliferation inhibition capacity, vascular endothelial growth factor secretion) should be incorporated as companion metrics in clinical trials. For veterinary clinical applications, establishing allogeneic MSC banks from healthy young donors should be prioritized to mitigate quality variability associated with autologous MSCs.

#### 7.2.3 Translational gap in pre-treatment strategies

Pre-treatment strategies significantly enhance MSC homing and therapeutic efficacy in rodent models, yet *in vivo* validation in dogs and cats remains largely unexplored. The most evidence-based pre-treatment method should be selected for *in vitro* potency validation in canine and feline MSCs (assessing CXCR4 expression, VEGF secretion, and anti-apoptotic capacity), followed by small-scale proof-of-concept trials to evaluate its potential to translate into improved clinical outcomes. Concurrently, attention must be paid to potential adverse effects of pre-treatment, such as conditions that may enhance one function at the expense of others.

#### 7.2.4 Feasibility exploration of minimally invasive local delivery

Although renal artery injection and subcapsular renal injection can elevate renal retention rates to 15–20% or higher in rodent models, their application in veterinary clinical practice is constrained by technical barriers, anesthesia risks, and owner acceptance. While safety has been demonstrated in porcine models, clinical trials involving cats and dogs are still required to conduct safety assessments, thereby providing robust data support for the widespread adoption of this technique.

### 7.3 Long-term development: next-generation therapies and scientific regulation

Exosomes (EVs) have emerged as a promising “cell-free” alternative to MSCs due to their low immunogenicity, high permeability, and amenability to engineering modification (159–163). However, challenges persist in EV isolation and purification, dose standardization, long-term safety, and efficacy maintenance. Future efforts should focus on establishing GMP-grade scaled production processes for EVs, completing toxicological and pharmacokinetic studies, and conducting safety trials in client-owned cats and dogs with naturally occurring disease. Concurrently, leveraging RGD-modified hydrogel delivery systems to enhance EV retention and internalization in damaged renal tubules, in parallel with developing engineered EVs, could enable targeted delivery and optimized cargo loading (164).

### 7.4 Validation pathway for the “pre-treatment enhancement–targeted delivery–carrier assisted” integration strategy

Based on the hierarchical analysis outlined above, future research should systematically validate the “Prime–Target–Carrier” framework proposed in this paper:

Prime (Pre-treatment Enhancement): Screen optimal pre-treatment protocols (e.g., hypoxia, melatonin, ATRA) in canine and feline MSCs, establish potency assay methods (CXCR4 expression, VEGF secretion, anti-apoptotic capacity), and conduct small-sample proof-of-concept trials.

Target (Targeted Delivery): Develop operational protocols and safe dosage ranges for ultrasound-guided subcapsular renal injection in large animal models, then apply the optimized protocol to animals with naturally occurring nephropathy to compare the efficacy of local versus intravenous administration.

Carrier (Vehicle-Assisted): Prioritize injectable, minimally invasive carriers (e.g., degradable hydrogels, microcapsules), conduct safety trials in dogs and cats, and evaluate owner acceptance and cost-effectiveness.

In summary, only through this problem-oriented, tiered research strategy—combined with systematic validation using the “Prime–Target–Carrier” framework—can MSC therapy transition from experimental treatment to routine clinical practice for companion animals.

## 8 Conclusion

This review summarizes research progress on mesenchymal stem cell therapy for both acute kidney injury and chronic kidney disease. Existing evidence indicates that MSC therapy is generally safe and well tolerated, with the potential to improve renal function and reduce histopathological damage. However, its clinical application remains constrained by low delivery efficiency, insufficient cell survival, and limited quality of evidence.

To achieve efficient MSC homing and enhance therapeutic efficacy, the integrated “Prime–Target–Carrier” strategy remains the focus of current optimization efforts. However, critical challenges persist across this framework. Optimal pre-treatment conditions remain unstandardized, clinical data on the risks versus benefits of local injection are lacking, and the feasibility of cellular carriers requires further evaluation.

Future research should advance using a phased approach: In the short term, establish product standards and conduct multicenter randomized controlled trials (RCTs). In the medium term, optimize delivery pathways, validate pre-treatment strategies, and utilize single-cell technology to analyze heterogeneity. In the long term, promote the translation of cell-free therapies and establish a veterinary regulatory framework. Concurrently, leveraging natural kidney disease models in cats and dogs to accumulate real-world evidence will help advance the safe and effective application of MSC therapies in companion animal clinical settings.
